# Supplementary material for: Prognostic Impact and Predictors of New-Onset Atrial Fibrillation in Heart Failure
Source: Life (Basel). 2022 Apr 13;12(4):579. doi: 10.3390/life12040579 (PMC9025044; doi:10.3390/life12040579)
Supplement: Supplementary file 1 [file life-12-00579-s001.zip › life-1662823-supplementary.pdf]

Supplementary Table S1. Baseline characteristics according to previous AF history

	AF history (+) (n = 2693)	AF history (-) (n = 2932)	**p value**
Demographics			
Age, year	70.9 ±12.8	66.3 ±15.6	< 0.001
Male (%).	1402 (52.1)	1591 (54.3)	0.10
BMI, kg/m^2	23.3 ±3.85	23.3 ±3.92	0.58
Current smoking (%)	371 (13.8)	622 (21.2)	< 0.001
Comorbidities			
Hypertension (%)	1632 (60.6)	1693 (57.7)	0.03
Diabetes mellitus (%)	828 (30.7)	1158 (39.5)	< 0.001
Chronic kidney disease (%)	365 (13.6)	440 (15.0)	0.13
Chronic obstructive lung disease (%)	345 (12.8)	288 (9.82)	< 0.001
Cerebrovascular accident (%)	497 (18.5)	356 (12.1)	< 0.001
Implantable cardioverter defibrillator (%)	49 (1.82)	37 (1.26)	0.11
Cardiac resynchronization therapy (%)	13 (0.48)	20 (0.68)	0.42
Etiology			
Ischemic heart disease (%)	669 (24.9)	918 (31.3)	< 0.001
Dilated cardiomyopathy (%)	223 (8.28)	250 (8.53)	0.78
Valvular heart disease (%)	577 (21.4)	231 (7.88)	< 0.001

	AF history (+) (n = 2693)	AF history (-) (n = 2932)	**p value**
Clinical status on admission			
Lung congestion	2132 (79.2)	2305 (78.6)	0.64
Heart failure category			< 0.001
De novo heart failure	1199 (44.5)	1737 (59.2)	
Acute decompensated heart failure	1494 (55.5)	1195 (40.8)	
Acute kidney injury	428 (15.9)	318 (10.8)	< 0.001
Systolic BP, mmHg	129 (28.8)	133 (31.4)	< 0.001
Diastolic BP, mmHg	77.9 (18.8)	79.2 (18.7)	0.01
Heart rate, beats/min	94.7 (29.3)	90.7 (22.3)	< 0.001
NYHA class (%)			0.005
II	369 (13.7)	486 (16.6)	
III	1032 (38.3)	1042 (35.5)	
IV	1292 (48.0)	1404 (47.9)	
Hemoglobin, g/dL	12.5 ± 2.24	12.3 ± 2.37	0.03
Creatinine, mg/dL	1.43 ± 1.31	1.54 ± 1.61	0.007
BUN, mg/dL	27.0 ± 16.7	25.6 ± 16.2	0.001
BNP, pg/mL	1172 ± 1211	1485 ± 1363	< 0.001
NT-proBNP, pg/mL	9088 ± 10158	9376 ± 11355	0.46

	AF history (+) (n = 2693)	AF history (-) (n = 2932)	**p value**
Electrocardiographic parameters			
RBBB (%)	213 (7.91)	185 (6.31)	0.02
LBBB (%)	103 (3.82)	191 (6.51)	< 0.001
Interventricular conduction disturbance (%)	170 (6.31)	177 (6.04)	0.71
Q wave (%)	312 (11.6)	430 (14.7)	0.001
PR interval, msec	173 ± 57.0	168 ± 37.8	0.06
QRS width, msec	106 ± 29.1	108 ± 33.2	0.03
QT interval, msec	378 ± 69.6	397 ± 60.6	< 0.001
Corrected QT interval, msec	473 ± 48.3	476 ± 45.2	0.01
Heart rate, beats/min	102 ± 31.5	91.0 ± 28.0	< 0.001
Echocardiographic parameters			
LV end-diastolic dimension, mm	56.3 ± 9.91	58.5 ± 10.2	< 0.001
LV end-systolic dimension, mm	43.7 ± 12.0	46.7 ± 12.5	< 0.001
LV EF, %	42.2 ± 15.2	38.9 ± 15.1	< 0.001
LA diameter, mm	51.5 ± 10.3	45.2 ± 8.26	< 0.001
Peak TR velocity, m/sec	2.90 ± 0.58	2.90 ± 0.61	0.99
Estimated RV systolic pressure, mmHg	44.3 ± 14.5	43.6 ± 15.7	0.13
Discharge medication			

	AF history (+) (n = 2693)	AF history (-) (n = 2932)	**p value**
RAS blocker (%)	1606 (59.6)	2102 (71.7)	< 0.001
Beta-blocker (%)	1204 (44.7)	1602 (54.6)	< 0.001
Aldosterone antagonist (%)	1230 (45.7)	1296 (44.2)	0.28
Nitrates (%)	695 (25.7)	54 (28.9)	0.38
Loop diuretics (%)	1935 (71.9)	2056 (70.1)	0.16
Thiazide (%)	236 (8.76)	221 (7.54)	0.10
Statin (%)	869 (32.3)	1459 (49.8)	< 0.001
Clinical status on discharge			
BMI, kg/m^2	23.3 ± 3.81	22.5 ± 3.83	0.60
Systolic BP, mmHg	114 ± 16.5	115 ± 18.4	0.07
Diastolic BP, mmHg	66.9 ± 11.4	67.3 ± 11.5	0.19
Heart rate, beats/min	76.0 ± 15.0	77.5 ± 13.4	< 0.001
NYHA class (%)			< 0.001
I	394 (14.6)	555 (18.9)	
II	1722 (63.9)	1955 (66.7)	
III	159 (5.90)	187 (6.38)	
IV	85 (3.16)	127 (4.33)	
Creatinine, mg/dL	1.34 ± 1.20	1.45 ± 1.49	0.004

	AF history (+)<br>(n = 2693)	AF history (-)<br>(n = 2932)	**p value**
BUN, mg/dL	25.3 ± 16.8	24.1 ± 15.1	0.004
BNP, pg/mL	1048 ± 1144	1281 ± 1290	< 0.001
NT-proBNP, pg/mL	8798 ± 9889	8683 ± 11147	0.76

NOAF, new-onset atrial fibrillation; BMI, body mass index; BP, blood pressure; NYHA, New York Heart Association; BUN, blood urea nitrogen; BNP, blood natriuretic peptide; NT-proBNP, N-terminal proBNP; RBBB, right bundle branch block; LBBB, left bundle branch block; LV, left ventricular; EF, ejection fraction; LA, left atrium; TR, tricuspid regurgitation; RV, right ventricular; RAS, renin-angiotensin system
